# Comorbidity and nutritional status in adult with advanced chronic kidney disease influence the decision-making choice of renal replacement therapy modality: A retrospective 5-year study

**DOI:** 10.3389/fnut.2023.1105573

**Published:** 2023-02-16

**Authors:** Graciela Álvarez-García, Ángel Nogueira Pérez, María Pilar Prieto Alaguero, Carmen Pérez Garrote, Aránzazu Díaz Testillano, Miguel Ángel Moral Caballero, Mar Ruperto, Cristina González Blázquez, Guillermina Barril

**Affiliations:** ^1^Department of Nephrology, Hospital Universitario de la Princesa, Madrid, Spain; ^2^Department of Pharmaceutical and Health Sciences, School of Pharmacy, Universidad San Pablo-CEU, CEU Universities, Madrid, Spain; ^3^Department of Nursing, School of Medicine, Universidad Autónoma de Madrid, Madrid, Spain

**Keywords:** advanced chronic kidney disease, comorbidity, Charlson comorbidity index, home-based renal replacement therapy, nutritional status, prognosis nutritional index, protein-energy wasting, renal replacement therapy

## Abstract

**Background:**

Nutritional and inflammation status are significant predictors of morbidity and mortality risk in advanced chronic kidney disease (ACKD). To date, there are a limited number of clinical studies on the influence of nutritional status in ACKD stages 4–5 on the choice of renal replacement therapy (RRT) modality.

**Aim:**

This study aimed to examine relationships between comorbidity and nutritional and inflammatory status and the decision-making on the choice of RRT modalities in adults with ACKD.

**Methods:**

A retrospective cross-sectional study was conducted on 211 patients with ACKD with stages 4–5 from 2016 to 2021. Comorbidity was assessed using the Charlson comorbidity index (CCI) according to severity (CCI: ≤ 3 and >3 points). Clinical and nutritional assessment was carried out by prognosis nutritional index (PNI), laboratory parameters [serum s-albumin, s-prealbumin, and C-reactive protein (s-CRP)], and anthropometric measurements. The initial decision-making of the different RRT modalities [(in-center, home-based hemodialysis (HD), and peritoneal dialysis (PD)] as well as the informed therapeutic options (conservative treatment of CKD or pre-dialysis living donor transplantation) were recorded. The sample was classified according to gender, time on follow-up in the ACKD unit (≤ 6 and >6 months), and the initial decision-making of RRT (in-center and home-RRT). Univariate and multivariate regression analyses were carried out for evaluating the independent predictors of home-based RRT.

**Results:**

Of the 211 patients with ACKD, 47.4% (*n* = 100) were in stage 5 CKD, mainly elderly men (65.4%). DM was the main etiology of CKD (22.7%) together with hypertension (96.6%) as a CV risk factor. Higher CCI scores were significantly found in men, and severe comorbidity with a CCI score > 3 points was 99.1%. The mean time of follow-up time in the ACKD unit was 9.6 ± 12.8 months. A significantly higher CCI was found in those patients with a follow-up time > 6 months, as well as higher mean values of eGFR, s-albumin, s-prealbumin, s-transferrin, and hemoglobin, and lower s-CRP than those with a follow-up <6 months (all, at least *p* < 0.05). The mean PNI score was 38.9 ± 5.5 points, and a PNI score ≤ 39 points was found in 36.5%. S-albumin level > 3.8 g/dl was found in 71.1% (*n* = 150), and values of s-CRP ≤ 1 mg/dl were 82.9% (*n* = 175). PEW prevalence was 15.2%. The initial choice of RRT modality was higher in in-center HD (*n* = 119 patients; 56.4%) than in home-based RRT (*n* = 81; 40.5%). Patients who chose home-based RRT had significantly lower CCI scores and higher mean values of s-albumin, s-prealbumin, s-transferrin, hemoglobin, and eGFR and lower s-CRP than those who chose in-center RRT (*p* < 0.001). Logistic regression demonstrated that s-albumin (OR: 0.147) and a follow-up time in the ACKD unit >6 months (OR: 0.440) were significantly associated with the likelihood of decision-making to choose a home-based RRT modality (all, at least *p* < 0.05).

**Conclusion:**

Regular monitoring and follow-up of sociodemographic factors, comorbidity, and nutritional and inflammatory status in a multidisciplinary ACKD unit significantly influenced decision-making on the choice of RRT modality and outcome in patients with non-dialysis ACKD.

## 1. Introduction

Chronic kidney disease (CKD) has become a major public health problem due to its high incidence, prevalence, and associated morbidity and mortality (1). From the early stages of CKD and as the estimated glomerular filtration rate (eGFR) progresses, cardiovascular (CV) risk often increases exponentially and constitutes the leading cause of mortality in patients with CKD (2). Epidemiological data from the Global Burden of Disease Study 2017 (3) have shown that CKD is one of the leading causes of death in the last few years. The aging population and the increasing trend of CKD risk factors jointly contributed to more than half of CKD deaths. The main etiology of CKD varies according to the setting with high blood pressure (HBP), and diabetes mellitus (DM) being the most frequent causes of CV risk and adverse prognosis (1, 3).

The guidelines for CKD from the National Kidney Foundation’s Kidney Disease Outcomes Quality Initiative (KDOQI) (4) and the Clinical Practice Guideline for the Evaluation and Management of Chronic Kidney Disease (KDIGO) (5) recommend stage 4 and depending on the etiology and rate of progression of CKD, and close to stage 5 (eGFR: < 15 ml/min/1.73 m^2^), it is mandatory to inform about the available therapeutic options including dialysis modalities. The educational approach is based on age, comorbidity, magnitude of proteinuria, and nutritional status among other clinical variables frequently assessed (5). There are usually different renal replacement therapy (RRT) modalities, including in-center hemodialysis (HD) and home-RRT [home-based HD, and peritoneal dialysis (PD)]. Other available therapeutic options in patients who choose not to start dialysis include conservative treatment of CKD and pre-dialysis living donor kidney transplantation. The K/DOQI guidelines (4) for the clinical evaluation, classification, and stratification of CKD recommend the assessment of the potential risks and benefits to make the most appropriate decision-making on when to start RRT.

Patients with advanced chronic kidney disease (ACKD) stage 4 or 5 have a severely decreased eGFR (<30 ml/min/1.73 m^2^) and are therefore candidates for intensive monitoring and care in the specialized ACKD unit (6). Multidisciplinary ACKD units aim to provide comprehensive care to prevent and/or treat associated comorbidities and improve the quality of life at the end stages of CKD. The clinical approach consists of early referral to a specialized unit for the management and follow-up of CKD by a nephrologist, at least 6 months before the onset of RRT (7). Late referral of patients to ACKD units is associated with increased adverse outcomes and reduced long-term overall survival from all causes (8). Lack of prior information and/or education about available therapeutic options in CKD contributes to reduced use of home-RRT modalities or living donor kidney transplantation, as well as promoting unplanned and urgent initiation of dialysis (9, 10).

Nutritional disorders are significantly associated with morbidity and mortality in patients with ACKD and dialysis (11, 12). Causative factors such as lack of appetite and insufficient dietary intake of energy and protein due to the dietary restrictions of CKD, metabolic disturbances as well as metabolic acidosis, or the detrimental effects of the inflammatory state significantly increase the nutritional risk in patients with ACKD (12).

The prognostic nutritional index (PNI) score is a new composite indicator that includes a combination of serum albumin (s-albumin) and total lymphocyte count (TLC) (13). Previous studies (14–18) demonstrated that the PNI is related to poor clinical outcomes and predicts survival in a variety of solid tumors, postoperative complications, and other disease states. A cut-off point of the PNI score < 39 points has been recognized as an independent prognostic marker of clinical and mortality outcomes in older patients with CKD (19, 20) and patients with dialysis (21, 22).

In 2008 (23), the *International Society for Renal Nutrition and Metabolism (ISRNM)* proposed the term protein-energy wasting (PEW) syndrome in CKD as more than insufficient food intake, including disturbances in biochemical markers such as s-albumin, body composition, and the contribution of comorbidities and underlying inflammation. The diagnosis of PEW is based on several categories in which biochemical markers (e.g., s-albumin), body mass index (BMI), muscle mass, and dietary protein intake when accompanied by inflammation are usually modified (23). PEW is a common disorder estimated in 28–54% of patients with non-dialysis CKD (24). A retrospective cross-sectional study in 307 patients with CKD (11) showed that previous nutritional follow-up time, serum prealbumin (s-prealbumin), and right-handgrip strength were independent predictors of mortality risk at 10-year follow-up. Early identification of patients at nutritional risk and the use of nutritional screening tools (e.g., PNI score) together with a combination of several nutritional markers are necessary to decide to initiate nutritional support.

Identification and assessment of modifiable factors, such as nutritional risk and PEW, as well as the management of the most common comorbid conditions, may be clinically useful in preventing and/or avoiding underlying complications in end-stages of CKD. Consequently, it seems important to assess modifiable risk factors (i.e., nutritional and inflammatory status), together with CV risk factors (DM, hypertension) and underlying comorbidities, before informing patients with ACKD about available CKD therapeutic options or initiating RRT. Achieving or maintaining adequate nutritional status is one of the goals and challenges in ACKD stages 4–5, as well as at the time of choosing the RRT modality or before starting dialysis. This study aimed to examine relationships between comorbidity and nutritional and inflammatory status and the decision-making on the choice of RRT modalities in adult patients with ACKD.

## 2. Materials and methods

### 2.1. Study design and study population

A retrospective cross-sectional observational study was carried out at the Hospital Universitario La Princesa (Madrid, Spain). Data were collected retrospectively from December 2016 to December 2021 on adult patients with ACKD who attended the multidisciplinary ACKD unit in the last 5 years. Participants were required to meet the following inclusion criteria: adults (18 years or over) patients with CKD in stages 4–5 [eGFR: ≤ 20 ml/min/1.73 m^2^] who choose any RRT modality [in-center (HD) and home-based (HD or peritoneal dialysis (PD)], conservative CKD treatment and pre-dialysis living donor transplant in the last 5 years at the ACKD unit. Patients with CKD stages 1–3b and those with an eGFR >30 ml/min/1.73 m^2^ were excluded from this study.

According to the KDIGO guidelines (5) is recommended to refer patients with eGFR <30 ml/min/1.73 m^2^ (eGFR stages: G4–G5) to a nephrologist specializing in ACKD, and to initiate information and education on RRT modalities and available treatment options.

This study was approved by the Ethics Committee of Hospital Universitario de La Princesa and was conducted according to the guidelines of the Declaration of Helsinki (Code number: 4247).

### 2.2. Data collection

The data collected and selected for the study coincided with the scheduled visit to inform about the different modalities of RRT (in-center, home-based HD, and PD) and informed therapeutic options available (conservative treatment of CKD or pre-dialysis living donor transplantation), within the framework of the clinical and care protocol of the ACKD unit.

The estimated glomerular filtration rate (eGFR) was measured by the Chronic Kidney Disease Epidemiology Collaboration (CKD-EPI) using the 2021 CKD-EPI Creatinine equation (25). The eGFR was classified according to the KDIGO clinical practice guidelines for the evaluation and management of CKD (5).

Sociodemographic data, laboratory parameters, and most frequent comorbidities were collected retrospectively from the medical record of each participant. The date of admission and discharge was retrospectively registered to define the mean follow-up time at the ACKD unit in the last 5 years. The sample was classified according to the median follow-up time using a cut-off point of 6 months to assess the influence of nutritional and inflammatory status as well as underlying comorbidities on clinical outcomes.

### 2.3. Assessment of comorbidity

The comorbidity was assessed using the modified Charlson comorbidity index (CCI) score (26). It consists of 19 items that include the most frequent pathologies or comorbid conditions and adds one point for each decade in patients aged 50 years and older. The sum of the CCI items classifies comorbidity as follows: no comorbidity (0 points), low comorbidity (1–2 points), and severe comorbidity (≥ 3 points) (26).

Blood pressure (BP) was measured using an automatic blood pressure monitor (OMROM®; M6; Netherlands, EU). The mean BP values corresponding to the three different measurements were recorded to improve the reproducibility of BP measurements as a standard procedure in the clinical practice of the multidisciplinary ACKD unit every 3 months coinciding with the scheduled medical visit. The mean blood pressure collected in this study coincides with the BP measured during the medical visit in which the RRT modalities. HBP was defined as systolic and diastolic BP ≥ 140/90 mmHg and was considered a CV risk factor (27).

### 2.4. Laboratory parameters

Biochemical and hematological parameters were retrospectively collected from medical records for all participants before choosing the RRT modality or therapeutic election. S-albumin (g/dl), s-prealbumin (mg/dl), serum transferrin (s-transferrin; mg/dl), and serum C-reactive protein (s-CRP; mg/dl), hemoglobin (Hb), and TLC concentrations were analyzed by automated standardized methods in the laboratory of the Hospital Universitario de La Princesa. All parameters were analyzed by the standard clinical protocol of the ACKD unit.

### 2.5. Assessment of nutritional and inflammatory status

The prognostic nutritional index (PNI) is a novel score that has previously been used under several disease conditions and patients with CKD (14–20). The PNI score was calculated as follows: [10 × serum albumin (g/dl) + (0.005 × total lymphocyte count (cells x 10^3^ mm^3^] (13). According to previously published studies (19, 20) and mean PNI values in the sample, the cut-off point of the PNI score was set at 39 points. Patients were classified into two groups according to the PNI score cut-off point as follows: nutritional risk (PNI: ≤ 39 points) and no nutritional risk (PNI: >39 points). Levels of the s-albumin <3.8 g/dl and s-CRP >1 mg/dl were used according to the criteria proposed by the ISRNM together with the PNI score to define nutritional risk and PEW (23).

As part of the standard management and care of patients with ACKD, nutritional status is assessed and monitored every 3 months at scheduled visits by a renal dietitian-nutritionist, or more frequently as required by the patient in the ACKD unit. Patients with ACKD receive regular and individualized nutritional counseling and medical follow-up. Protein intake of 0.6–0.8 g/kg/day is recommended in patients with diabetic CKD and 0.5–0.6 g/kg/day in patients with non-diabetic CKD, together with a salt-free diet and low potassium and phosphorus intake (28). An individualized energy intake between 25 and 35 kcal/kg/day based on age, gender, physical activity level, body composition, weight goals, CKD stage, and concurrent comorbidities or the presence of inflammation or other metabolic disturbances is usually recommended to achieve and/or maintain adequate nutritional status according to KDOQI Guidelines on Nutrition (28). Nutritional recommendations and the individualized diet are personalized according to the stage of CKD, laboratory parameters, and the patient’s progress at each of the scheduled medical visits. Nutritional management is usually carried out in collaboration with a multidisciplinary team (a nephrologist, a nurse specializing in Nephrology, and a dietitian–nutritionist).

### 2.6. Statistical analysis

The sample size was calculated using the statistical program G. Power version 3.1.9.4 (Franz Faul, Universitat Kiel, Germany) with a power of 90% and a significance level of 5%. The study required a sample size of 137 subjects to detect significant interactions with the RRT modality. The effect size was calculated using Cohen’s *d*-value according to RRT modality (in-center or home-based) and the mean age of each group. Cohen’s *d*-value was 0.723, and the calculated effect size was 0.345. Data are expressed as mean ± standard deviation and as frequencies or percentages according to the nature of the variable analyzed. To compare the frequency and mean differences, *p*-values were calculated using Chi-square and Fisher’s exact test for categorical variables and Student’s *t*-test or the non-parametric Mann–Whitney U-test for continuous variables. Pearson’s Chi-square parametric correlations were examined to assess the strength of the association between the variables. Univariate and multivariate logistic regression analyses were used, and the corresponding odds ratio (OR) and 95% confidence interval (95%CI) were calculated. In-center and home-based dialysis modalities were used as the dependent and dichotomized variables in the univariate and multivariate regression analyses. Only data from the univariate analysis that had a value of *p* of 0.10 or less were tested *a priori* to explore possible changes in the response variable during multiple logistic regression analysis. A binary logistic regression model using the forward stepwise conditional method was used. The Statistical Package for Social Science (SPPS for Windows) version 23.0 was used in all statistical analyses. A value of *p* of <0.05 was considered statistically significant.

## 3. Results

### 3.1. General characteristics of the study population and classification according to gender

Table 1 summarizes clinical and biochemical parameters characteristics in the study population and according to gender. Of the 211 patients with ACKD, 138 patients with CKD were men (65.4%), mainly older than women (*p* = 0.020). A higher men proportion were living with a family, had a university education, and were active workers (Table 1). The mean value of eGFR was 13.7 ± 3.4 ml/min/1.73 m^2^. According to the eGFR staging, 52.6% (*n* = 111) were in stage 4 CKD and 47.4% (*n* = 100) were in stage 5 CKD. In CKD stage 4, 68.4% (*n* = 76) were men, while in CKD stage 5, 62.0% (*n* = 62) were men. Mean eGFR values did not differ significantly between men and women (*p* = 0.183).

**Table 1:** Clinical and biochemical parameters characteristics of 211 participants in the study and by gender.

Variables	Total (*n* = 211)	Male (*n* = 138)	Female (*n* = 73)
Age (years)	71.7 ± 12.8	73.6 ± 11.2	68.1 ± 15.0
Living with a family *n* (%)	138 (65.4)	105 (76.1)	33 (45.2)
Active workers *n* (%)	65 (30.8)	40 (28.9)	25 (34.2)
University education *n* (%)	73 (34.6)	50 (36.2)	23 (31.5)
eGFR (mL/min/1.73 m^2^)^*^	13.7 ± 3.4	14.0 ± 3.5	13.3 ± 3.1
CCI (points) CCI: ≤ 3 points *n* (%) CCI: >3 points *n* (%)	6.5 ± 1.3 2 (0.9) 209 (99.1)	6.8 ± 1.1 1 (0.7) 137 (99.2)	6.1 ± 1.4 1 (1.3) 72 (98.6)
DM *n* (%)	48 (22.7)	32 (23.1)	16 (21.9)
High blood pressure *n* (%)	204 (96.6)	132 (95.6)	72 (98.6)
Time on follow-up in ACKD unit (months)	9.6 ± 12.8	9.2 ± 12.3	10.3 ± 13.7
s-Albumin (g/dl)	3.8 ± 0.5	3.9 ± 0.5	3.8 ± 0.5
s-Prealbumin (mg/dl)	28.9 ± 5.1	29.0 ± 5.4	28.7 ± 4.3
s-Transferrin (mg/dl)	200.4 ± 49.1	198.7 ± 42.1	203.6 ± 60.5
s-CRP (mg/dl)	0.5 ± 1.1	0.6 ± 1.2	0.5 ± 0.9
Hemoglobin (g/dl)	11.2 ± 1.23	11.2 ± 1.2	11.2 ± 1.1
Total lymphocyte count (10^3^/mm^3^)	2,035 ± 0.4	2,065 ± 0.4	1,978 ± 0.4
PNI (points)	38.9 ± 5.5	38.8 ± 5.7	39.1 ± 5.2

CCI, Charlson comorbidity index; PNI, prognostic nutritional index; s-CRP, serum C-reactive protein.

* eGFR, estimated glomerular filtrate rate was measured by the 2021 CKD-EPI equation (25).

Mean CCI score values were 6.5 ± 1.3 points, with significantly higher scores found in men (6.8 ± 1.1 points) than in women (6.1 ± 1.4 points; *p* < 0.001). Analyzing the CCI score, 99.10% (*n* = 209) had severe comorbidity (CCI: > 3 points), while only two patients with ACKD (0.9%) had low comorbidity (CCI: ≤ 3 points). DM was the main diagnosed cause leading to CKD in 22.7% (*n* = 48). HBP accounted for 96.6% (*n* = 204) and was significantly more frequent in men (*n* = 132; 95.6%) than in women (*n* = 72; 98.6%). Other commonly associated comorbidities were peripheral vascular disease and cerebrovascular disease. The mean time of medical follow-up in the ACKD unit was 9.6 ± 12.8 months with no significant differences between men and women (*p* = 0.56; Table 1).

Mean s-albumin values were 3.8 ± 0.5 g/dl without significant differences between gender. The cut-off point of s-albumin level > 3.8 g/dl was found in 71.1% (*n* = 150) of patients with ACKD, more often in men (45.5%; *n* = 54) than in women (25.6%; *n* = 54; *p* = 0.30; data not shown). Mean values of s-CRP were 0.5 ± 1.1 mg/dl without non-significant differences between gender (*p* = 0.66). Values of s-CRP ≤ 1 mg/dl were found in 175 patients with ACKD (82.9%) in a similar way in men (82.6%) than in women (83.6%; *p* = 0.51; data not shown). The conjoint use of the cut-off points of s-albumin <3.8 g/dl and s-CRP ≥ 1 mg/dl was found in 15.2% (*n* = 32) as PEW markers, being more frequent in men (10.4%; *n* = 22). No significant differences were found with biochemical markers such as s-prealbumin, s-transferrin, or hematological parameters (hemoglobin, TLC) between both groups (Table 1).

The mean PNI score was 38.9 ± 5.5 points and was found to be similar between men (PNI: 38.8 ± 5.7 points) and women (PNI: 39.1 ± 5.2 points) subjects (*p* = 0.707). A cut-off point of PNI ≤ 39 points was found in 77 adults with ACKD (36.5%) with a non-significant higher frequency in the male group than in the female group (68.8 vs. 32.2%; *p* = 0.45; Table 1).

### 3.2. Comparison of clinical and biochemical parameters characteristics in the study according to the previous follow-up time in the advanced chronic kidney disease unit

Table 2 shows the results according to follow-up time in the ACKD unit. The median follow-up time was 6 months (r: 1–64 months). No significant differences were found with gender, living with a family, having university studies, and being an active worker with the follow-up time in the ACKD unit (Table 2). Patients with ACKD on follow-up time > 6 months accounted for 42.1% (*n* = 89). Higher CCI was significantly found in those patients with follow-up time > 6 months (CCI score: 7.1 ± 1.1 points) than those who had a follow-up time ≤ 6 months (CCI: 6.3 ± 1.3 points; *p* = 0.024). CCI score was >3 points in more than 98.0% of patients with ACKD follow-up in the ACKD unit. No differences were found in patients with diabetic and hypertensive ACKD according to the previous follow-up time. Notably, patients with regular follow-up for more than 6 months had significantly higher mean values of eGFR, s-albumin, s-prealbumin, s-transferrin, and hemoglobin, and lower levels of s-CRP than those with follow-up ≤6 months (*p* < 0.001). Mean PNI values were significantly higher in patients with ACKD with follow-up time > 6 months (Table 2). Only 13 patients with ACKD (18.8%) with a follow-up time > 6 months had a PNI score ≤ 39 points compared with those with follow-up time in the ACKD unit ≤6 months (81.2%; *n* = 53; *p* < 0.001; data not shown).

**Table 2:** Comparison of clinical and biochemical parameters of 211 participants in the study according to the time on follow-up in the ACKD unit.

Variables	No follow-up ACKD unit ≤6 months (*n* = 122)	Follow-up ACKD unit >6 months (*n* = 89)	*p*-Value
Age (years)	67.6 ± 14.5	72.7 ± 12.3	0.050
Male *n* (%)	83 (68.0)	55 (61.7)	0.381
Female *n* (%)	39 (31.9)	34 (38.2)	
Living with a family *n* (%)	60 (49.1)	61 (68.5)	0.600
Active workers *n* (%)	23 (18.8)	30 (33.7)	0.565
University education *n* (%)	33 (27.0)	34 (38.2)	0.970
eGFR (mL/min/1.73 m^2^)^*^	12.8 ± 3.7	15.2 ± 2.3	<0.001
CCI (points) CCI: ≤ 3 points *n* (%) CCI: >3 points *n* (%)	6.3 ± 1.5 2 (1.6) 120 (98.3)	7.09 ± 1.1 0 89 (100%)	0.024
DM *n* (%)	28 (58.3)	20 (41.6)	0.991
High blood pressure *n* (%)	118 (96.7)	86 (96.6)	0.971
s-Albumin (g/dl)	3.7 ± 0.6	4.0 ± 0.4	0.002
s-Prealbumin (mg/dl)	27.5 ± 5.3	30.7 ± 4.1	<0.001
s-Transferrin (mg/dl)	188.3 ± 50.3	217.0 ± 42.5	<0.001
s-CRP (mg/dl)	0.8 ± 1.4	0.2 ± 0.3	<0.001
Hemoglobin (g/dl)	10.9 ± 1.4	11.6 ± 0.9	<0.001
Total lymphocyte count (10^3^/mm^3^)	2,064 ± 0.4	1,995 ± 0.4	0.296
PNI (points)	37.9 ± 6.0	40.3 ± 4.4	<0.001

ACKD, advanced chronic kidney disease. CCI, Charlson comorbidity index; PNI, prognostic nutritional index; s-CRP, C-reactive protein.

* eGFR, estimated glomerular filtrate rate was measured by the 2021 CKD-EPI equation (25).

### 3.3. Assessment of demographic, comorbidity, and biochemical parameters of the study population about the initial decision-making choice of in-center or home-based renal replacement therapy

Table 3 shows the comparison of clinical and biochemical parameters according to the free decision-making and initial choice of RRT. The initial choice of RRT modality was in-center HD (*n* = 119 patients; 56.4%) and home-based RRT (*n* = 81; 40.5%) in both home HD and PD modalities. Home-based HD accounts for 4.3%. Regarding the election of PD techniques, 44 patients (61.0%) chose continuous ambulatory PD and 28 patients with ACKD (38.8%) chose automated nocturnal peritoneal dialysis. Conservative treatment of CKD accounted for 4.3% (*n* = 9 patients), whereas pre-dialysis living donor transplantation was 0.9% (*n* = 2 patients).

**Table 3:** Comparison of clinical and biochemical parameters of 211 participants in the study according to the decision-making choice of home-based or in-center renal replacement therapy.

Variables	In-center RRT (*n* = 119)	Home-based RRT (*n* = 81)	*p*-Value	
Age (years)	74.9 ± 14.4	65.4 ± 11.3	<0.001	
Gender Male *n* (%) Female *n* (%)	79 (66.3) 40 (33.6)	53 (65.4) 28 (34.5)	0.889	
Living with a family *n* (%)	70 (58.8)	58 (71.6)	0.098	
Active workers *n* (%)	17 (14.3)	46 (56.8)	<0.001	
University education *n* (%)	19 (15.9)	53 (59.6)	<0.001	
eGFR (mL/min/1.73 m^2^)^*^	13.1 ± 3.7	14.6 ± 2.9	0.013	
CCI (points) CCI: < 3 points *n* (%) CCI: ≥ 3 points *n* (%)	6.9 ± 1.5 0 119 (100)	5.8 ± 11.1 2 (2.4) 79 (97.5)	0.163	
DM *n* (%)	34 (70.3)	14 (29.1)	0.990	
High blood pressure *n* (%)	117 (98.3)	77 (95.0)	0.225	
Time on follow-up in ACKD unit (months)	8.2 ± 12.6	11.1 ± 12.8	0.128	
s-Albumin (mg/dl)	3.7 ± 0.5	4.2 ± 0.4	<0.001	
s-Prealbumin (mg/dl)	27.3 ± 4.0	31.6 ± 5.6	<0.001	
s-Transferrin (mg/dl)	191.1 ± 52.2	218.2 ± 39.9	<0.001	
s-CRP (mg/dl)	0.8 ± 1.4	0.2 ± 0.5	<0.001	
Hemoglobin (g/dl)	10.8 ± 1.2	11.9 ± 1.0	<0.001	
Total lymphocyte count (10^3^/mm^3^)	2,099.0 ± 0.4	1,947.0 ± 0.4	0.795	
PNI (points)	37.2 ± 5.3	41.8 ± 4.5	<0.001	

CCI, Charlson comorbidity index; s-CRP, C-reactive protein; PNI, prognostic nutritional index.

* eGFR, estimated glomerular filtrate rate was measured by the 2021 CKD-EPI equation (25).

In-center RRT was chosen by patients with older, less occupationally active ACKD, with less university education and a mean CCI score of 6.9 ± 1.1 points (*p* < 0.001). Patients who chose home-based RRT had significantly lower CCI scores than those who chose in-center RRT (p < 0.001). In addition, patients who chose home-RRT had also significantly higher mean values of s-albumin, s-prealbumin, s-transferrin, hemoglobin, and eGFR and lower s-CRP than those who chose in-center RRT (p < 0.001). No significant differences were found with TLC between both groups (*p* = 0.79; Table 3).

### 3.4. Univariate and multivariate regression analyses

Table 4 shows the factors associated with the likelihood of choosing in-center or home-based RRT using a univariate binary regression analysis. Sociodemographic factors such as age, being active workers, and university education were significantly related to both in-center or home-based RRT choices. Higher levels of eGFR, s-albumin, s-prealbumin, s-transferrin, hemoglobin, and follow-up time in the ACKD unit >6 months were also significantly and independently associated with the free decision-making to choose in-center and home-based RRT modality (Table 4).

**Table 4:** Univariate binary regression analysis of factors associated with the decision-making to choose in-center or home-based renal replacement therapy.

Variable	In-center RRT OR beside (95% CI)	*p*-Value	Home-based RRT OR beside (95% CI)	*p*-Value
Age (years)	1.093 (1.060–1.128)	<0.001	1.095 (1.061–1.130)	<0.001
Gender (Male)	0.929 (0.512–1.685)	0.800	0.874 (0.483–1.583)	0.657
Living with a family (%)	0.531 (0.288–0.977)	0.042	0.567 (0.309–1.038)	0.066
Active workers (%)	0.089 (0.042–0.188)	<0.001	0.086 (0.041–0.182)	<0.001
University education (%)	0.095 (0.005–0.188)	<0.001	0.100 (0.051–0.196)	<0.001
eGFR (mL/min/1.73 m^2^)^*^	0.923 (0.848–1.004)	0.062	0.916 (0.841–0.997)	0.043
CCI (points)	2.973 (2.093–4.224)	<0.001	3.016 (2.117–4.297)	<0.001
Follow-up ACKD unit ≥6 months	0.807 (0.437–1.488)	0.492	0.539 (0.303–0.959)	0.036
s-Albumin (g/dl)	0.134 (0.062–0.291)	<0.001	0.107 (0.047–0.242)	<0.001
s-Prealbumin (mg/dl)	0.818 (0.756–0.884)	<0.001	0.819 (0.757–0.885)	<0.001
s-Transferrin (mg/dl)	0.989 (0.981–0.995)	<0.001	0.988 (0.982–0.995)	<0.001
s-CRP (mg/dl)	2.548 (1.434–4.526)	<0.001	2.603 (1.459–4.644)	<0.001
Hemoglobin (g/dl)	0.476 (0.346–0.654)	<0.001	0.476 (0.347–0.653)	<0.001
Total lymphocyte count (10^3^/mm^3^)	1.936 (1.038–3.612)	0.038	1.993 (1.068–3.719)	0.030
PNI (points)	1.229 (1.131–1.328)	<0.001	2.747 (1.500–5.032)	<0.001

CCI, Charlson comorbidity index; PNI, prognostic nutritional index; s-CRP, serum C-reactive protein.

* eGFR, estimated glomerular filtrate rate was measured by the 2021 CKD-EPI equation (25).

Multivariate binary logistic regression showed that well-known predictors such as s-albumin (OR: 0.147; 95% CI: 0.057–0.378) and follow-up time in the ACKD unit for >6 months (OR: 0.440; 95% CI: 0.204–0.950) were significantly related to the probability of choosing home-based RRT (all at least, *p* < 0.05), while age (OR: 1.570; 95% CI: 1.009–1.108) and CCI score (OR: 1.986; 95% CI: 1.251–3.154) were inversely associated with the probability of choosing home-based RRT (Table 5).

**Table 5:** Multivariate binary regression analysis of factors associated with the decision-making to choose home-based renal replacement therapy.

Variable	OR (95% CI)	Value of p
Age (years)	1.570 (1.009–1.108)	0.020
CCI (points)	1.986 (1.251–3.154)	0.004
Follow-up at ACKD unit >6 months	0.440 (0.204–0.950)	0.036
s-Albumin (g/dl)	0.147 (0.057–0.378)	<0.001

ACKD, advanced chronic kidney disease; CCI, Charlson comorbidity index.

## 4. Discussion

Study results demonstrate that certain sociodemographic factors (advanced age, living with a family, being an active worker and university education, as well as previous follow-up time in a multidisciplinary ACKD unit, and regular nutritional monitoring) with comorbidity status influenced the initial decision-making choice of RRT modality before starting dialysis in adults with ACKD stages 4–5. In this sample, patients with ACKD were older adults, were more often men, and were mainly in CKD stage 5 (62.0%). DM was the main etiology leading to CKD (22.7%) along with hypertension, which accounted for 96.6% were the most prevalent comorbidities found in patients with ACKD (Table 1).

Comorbidity as measured by CCI is associated with adverse outcomes and is a strong predictor of mortality in patients with dialysis (29, 30). A Canadian study of 530,771 patients with CKD highlighted that a higher degree of comorbidity was associated with worse outcomes, such as hospitalization, a longer length of hospital stay, and all-cause mortality (31). To date, there is a lack of studies on how comorbidity influences the decision-making choice of RRT modalities in adults with ACKD before starting dialysis. In this study, severe comorbidity significantly accounted for 99.1% of patients with ACKD. In fact, univariate and multivariate regression analyses significantly showed that the CCI score was inversely related to the probability of choosing home-based RRT. Due to the importance and complexity of this decision-making process, the importance of the multidisciplinary team is essential to support patients in their diagnosis and the complex decision about the initiation of dialysis (32).

Assessment and medical follow-up of underlying comorbidities related to CKD help to individualize and improve care in the setting of multidisciplinary ACKD units (33). In this study, it should be noted that a follow-up time > 6 months in the ACKD unit significantly improved eGFR, visceral protein profile (s-albumin, s-prealbumin, s-transferrin), s-CRP concentration, and mean hemoglobin levels in multimorbidity patients with ACKD (Table 2). A comprehensive clinical approach together with a follow-up time of >6 months significantly improved clinical outcomes as has been shown in the univariate and multivariate regression analyses of the study (Tables 4 and 5). Current strategies for the management of adults with ACKD indicate that a coordinated approach and intervention on modifiable risk factors through integrated and specialized care in the ACKD unit delays the progression of CKD and prevents complications and comorbidities before the onset of RRT (32, 34). The presence of specialized care programs before initiating dialysis and early education has been associated with increased adherence to treatment and dietary prescription (35). These assumptions mentioned earlier hold in the management of patients with ACKD, which increases the incidence of patient choice of home-RRT modalities and improves patients’ perception of autonomy (35, 36). Unscheduled initiation of dialysis and patients’ choice of initial RRT modality may also affect patients’ experiences and clinical outcomes (6, 33). Patients who are not referred early enough to a multidisciplinary nephrology team-led follow-up program, and unscheduled initiation of dialysis, are associated with increased morbidity and decreased survival in any RRT technique (9, 10).

Nutritional disorders are a common condition in ACKD associated with multimorbidity and worse survival outcomes (12). The PNI score is an indicator of immune and nutritional status that has been shown as an independent predictive risk factor in different disease conditions (14–18) as well as in older patients with ACKD (19, 20). A case–control study in older patients with ACKD stages 4–5 demonstrates that the median PNI score value was 48.37 points in an elderly Mediterranean cohort of patients with ACKD who had an adequate nutritional status when compared to age-sex matched with their controls (20). A PNI score < 39 points was a significant predictor of nutritional risk in patients with CKD stages 3–4 and has been associated with the early onset of RRT and an increased mortality rate (19). In this study, a PNI score ≤ 39 points as an indicator of nutritional risk was found in 36.5% of patients with ACKD stages 4–5, as well as a risk factor in the choice of RRT modality in the univariate analysis, in line with previously published studies (19, 20).

Protein-energy wasting is related to mild to moderate inflammatory states, favoring the progression of CKD and even accelerating early entry into RRT. S-albumin and s-CRP are well-known risk factors for morbidity and mortality in both patients with CKD and dialysis (11, 37, 38). In addition to the PNI risk score, PEW was measured using a combination of two biochemical markers s-albumin and s-CRP in agreement with ISRNM proposed PEW diagnosis criteria (23). It is noteworthy that the mean values of s-albumin and s-CRP in this study were following the normal ranges. Mostly, s-albumin level > 3.8 g/dl was found in 71.1% of the sample, while s-CRP ≤ 1 mg/dl was present in 175 patients with ACKD (82.9%; data not shown). In this study, s-albumin and s-CRP were significantly associated in the univariate analysis with the likelihood of choosing a home-based RRT (Table 4). However, in the multivariate regression analysis, only s-albumin was significantly related to the likelihood of choosing home-RRT (OR: 0.14; CI95%: 0.057–0.378; *p* < 0.001; Table 5). PEW prevalence measured by both biomarkers (s-albumin and s-CRP) was 15.1%. These results show a lower prevalence of PEW when compared with a previously published meta-analysis (24) in which PEW was 28–54% of patients with CKD. The possible divergence of results between the PNI score and the combination of PEW markers employed is partially linked to the sensitivity and cut-off points of the markers used, as well as the high mean s-albumin levels and low degree of inflammation found in this sample.

The KDIGO Clinical Guidelines (5) recommend initiating an informed scheme on the different RRT modalities and therapeutic options available in patients with stage 4 CKD. In the current study, the different modalities of RRT (in-center, home-based) along with other therapeutic options (conservative treatment of CKD and living donor transplantation in pre-dialysis) are usually informed by a nurse specialized in nephrology in the framework of standard care at the ACKD unit. Data results from this study showed that the initial decision-making choice was higher in-center RRT compared with home-based RRT modalities (Table 3), whereas conservative CKD treatment and pre-dialysis living donor transplantation were in both <5% in the sample. Patients with ACKD who chose home-RRT were mainly younger male subjects, more labor-active, with a higher level of university education and a lower degree of comorbidity compared to in-center RRT. Moreover, patients with ACKD who chose in-center RRT had significantly higher mean values of s-albumin, s-prealbumin, s-transferrin, and hemoglobin concentrations, as well as a lower degree of inflammation, as measured by mean s-CRP levels (Table 3). Nutritional risk measured by PNI score was significantly lower in patients who chose home-based RRT (18.8%) compared with in-center RRT (81.2%). Conversely, older age, s-CRP, and PNI scores ≤39 points were significant independent predictors in the univariate analysis for decision-making in-center and home-based RRT choices in adults with ACKD. Furthermore, follow-up time > 6 months, higher eGFR, and improvement of s-albumin, s-prealbumin, s-transferrin, and levels of hemoglobin concentrations (at least, *p* < 0.05) were also significantly associated with free decision-making of RRT (Table 4). These results are relevant from the perspective that clinical outcomes influence the patient’s decision to choose a home-based RRT modality. In addition, home-based RRT has been shown to have a positive impact on patient autonomy, quality of life, and health system costs (39, 40). Previous studies (32, 36) reported that patients with home-based RRT maintained independence and autonomy to work or study full-time as has been shown in the current study, and had also a better quality of life than those receiving HD at the center.

This study has some strengths and weaknesses that should be taken into account. This cross-sectional study is limited by the fact that it was conducted in a single ACKD unit, and the majority were older with a high prevalence of DM and severe comorbidity. Consequently, the results cannot be generalized to patients with early stages of CKD or in dialysis. However, the sample size of this study is relatively large. To the best of our knowledge, this is one of the few published studies that jointly assess the influence of comorbidity and nutritional status on the decision-making of choice of RRT modalities. By contrast, ISRNM protein-energy wasting criteria is a criterion to be assessed in future. Given the retrospective nature of the study, certain variables such as iron and lipid profiles, usual pharmacological treatment, dietary intake, body composition measurements, or quality of life before initiating any RRT modality were not recorded. Based on the earlier results, further longitudinal studies assessing the quality of life and mortality of adults with ACKD after admission to RRT seem relevant for future research.

## 5. Conclusion

In conclusion, regular monitoring and follow-up of sociodemographic factors, comorbidity, and nutritional and inflammatory status in a multidisciplinary ACKD unit significantly influenced decision-making on the choice of RRT modality in patients with non-dialysis ACKD. Early referral and follow-up >6 months in the ACKD unit improves clinical outcomes. Nutritional monitoring and follow-up of the patient together with underlying comorbidities help to identify and/or prevent potential CKD-related risk factors and to plan in advance nutritional intervention strategies before starting RRT. Further studies are required to evaluate longitudinally the impact of multimorbidity, nutritional, and inflammatory status on CKD progression.

## Data availability statement

The raw data supporting the conclusions of this article will be made available by the authors, without undue reservation.

## Ethics statement

This study was approved by the Ethics Committee of the Hospital Universitario de La Princesa, (Madrid, Spain) with code number: 4247. Written informed consent for participation was not required for this study in accordance with the national legislation and the institutional requirements.

## Author contributions

GÁ-G and GB developed the study design and methodology, research and data collection. ÁN, MC, and GÁ-G carried out the main analysis with supervision from GB and CB. In addition, GÁ-G and MR wrote the first and final drafts of the manuscript. GB, CB, MA, CG, AT, and MR made important critical revisions. Each author contributed important intellectual content during manuscript drafting. All authors contributed to the article and approved the submitted version.

## Conflict of interest

The authors declare that the research was conducted in the absence of any commercial or financial relationships that could be construed as a potential conflict of interest.

## Publisher’s note

All claims expressed in this article are solely those of the authors and do not necessarily represent those of their affiliated organizations, or those of the publisher, the editors and the reviewers. Any product that may be evaluated in this article, or claim that may be made by its manufacturer, is not guaranteed or endorsed by the publisher.
